# Phylogenetic Impoverishment of Amazonian Tree Communities in an Experimentally Fragmented Forest Landscape

**DOI:** 10.1371/journal.pone.0113109

**Published:** 2014-11-19

**Authors:** Bráulio A. Santos, Marcelo Tabarelli, Felipe P. L. Melo, José L. C. Camargo, Ana Andrade, Susan G. Laurance, William F. Laurance

**Affiliations:** 1 Departamento de Sistemática e Ecologia, Universidade Federal da Paraíba, João Pessoa, Paraíba, 58051-900, Brazil; 2 Departamento de Botânica, Universidade Federal de Pernambuco, Recife, Pernambuco, 50670-901, Brazil; 3 Biological Dynamics of Forest Fragment Project, National Institute for Amazonian Research (INPA), C.P. 478, Manaus, Amazonas, 69011-970, Brazil; 4 Centre for Tropical Environmental and Sustainability Science (TESS) and School of Marine and Tropical Biology, James Cook University, Cairns, Queensland, 4878, Australia; East China Normal University, China

## Abstract

Amazonian rainforests sustain some of the richest tree communities on Earth, but their ecological and evolutionary responses to human threats remain poorly known. We used one of the largest experimental datasets currently available on tree dynamics in fragmented tropical forests and a recent phylogeny of angiosperms to test whether tree communities have lost phylogenetic diversity since their isolation about two decades previously. Our findings revealed an overall trend toward phylogenetic impoverishment across the experimentally fragmented landscape, irrespective of whether tree communities were in 1-ha, 10-ha, or 100-ha forest fragments, near forest edges, or in continuous forest. The magnitude of the phylogenetic diversity loss was low (<2% relative to before-fragmentation values) but widespread throughout the study landscape, occurring in 32 of 40 1-ha plots. Consistent with this loss in phylogenetic diversity, we observed a significant decrease of 50% in phylogenetic dispersion since forest isolation, irrespective of plot location. Analyses based on tree genera that have significantly increased (28 genera) or declined (31 genera) in abundance and basal area in the landscape revealed that increasing genera are more phylogenetically related than decreasing ones. Also, the loss of phylogenetic diversity was greater in tree communities where increasing genera proliferated and decreasing genera reduced their importance values, suggesting that this taxonomic replacement is partially underlying the phylogenetic impoverishment at the landscape scale. This finding has clear implications for the current debate about the role human-modified landscapes play in sustaining biodiversity persistence and key ecosystem services, such as carbon storage. Although the generalization of our findings to other fragmented tropical forests is uncertain, it could negatively affect ecosystem productivity and stability and have broader impacts on coevolved organisms.

## Introduction

The current biodiversity crisis driven by human-induced species loss is likely to drastically alter the tree of life [1], [2], but this is still poorly understood due to the historical gap between ecology and phylogenetics [3]. Fortunately, this gap is shrinking as evolutionary relationships among species can be incorporated into ecological studies [4]–[6]. Phylogenetic information, besides providing an additional measure of biodiversity beyond traditional measures of species richness and diversity, is useful to infer community assembly processes and ecosystem stability [7]–[9].

In the context of conservation, community phylogenetics is a powerful tool to understand how biological communities respond to human disturbances of varying type, intensity, and frequency [10]. Important advances in this field are helping us to understand the effects of fires on community assembly [11]–[13], the processes involved in community organization during forest regeneration [14], [15], and the outcome of species loss and gain on community phylogenetic diversity [16], [17]. However, we are still far from drawing general conclusions about the organization and persistence of biodiversity in our rapidly changing world [18].

Several studies have revealed shifts in the taxonomic and functional profile of tropical tree communities in response to habitat loss and fragmentation [19]–[22]. Compared to continuous forest, tree communities near forest edges and in small forest fragments (hereafter edge-affected habitats), may support only a small fraction of (1) emergent and understory tree species; (2) heavy-wooded, shade-tolerant and slow-growing trees; (3) large-seeded trees dispersed by medium to large-bodied frugivores; and (4) tree species pollinated by specialized vectors and those bearing supra-annual reproduction (see Tabarelli et al. [23] and references therein). Long-term tree monitoring in Central Amazonia reveals that edge-affected habitats are hyperdynamic systems characterized by increased rates of tree recruitment and mortality, especially in the first few decades following edge creation [24]. This hyperdynamism is guided by the remarkable recruitment of fast-growing, early-to-mid successional trees that replace slow-growing late-successional ones [19], [25], [26], which are most probably killed by increased wind turbulence or desiccation near fragment edges [27], [28]. Similarly, in the Atlantic forest of northeast Brazil, a small number of native, fast-growing successional species proliferate from forest edges to entire landscapes [21], [29], leading to a sort of regional biotic homogenization [23], [30].

However, important questions remain before we can properly predict the destiny of tropical tree communities in human-modified landscapes. Two of them are addressed here: (1) how much evolutionary diversity is being lost in edge-affected habitats along with the taxonomic and functional impoverishment? (2) what is the role of human-induced biotic homogenization on the phylogenetic diversity of remaining communities? A recent study on secondary forests of Costa Rica [31] demonstrated that disturbance-favored early-successional tree species are more phylogenetically related than expected by chance, whereas late-successional species tend to be less related than random expectations drawn from the same regional phylogeny. If these trends hold for Amazonian tree species, we could expect an increase in phylogenetic clustering or a decrease in phylogenetic evenness in the edge-affected habitats, where fast-growing trees are thriving and replacing disturbance-sensitive ones.

In this paper we used one of the largest datasets currently available on tree dynamics in a 1000-km^2^ experimentally fragmented Amazonian forest landscape and a recent phylogeny of angiosperms to assess whether tree communities have lost phylogenetic diversity and become phylogenetically clustered since their isolation two decades previously. First, we examined the effects of forest site (1-ha, 10-ha, and 100-ha fragments and continuous forest), time since forest isolation, and distance to nearest forest edge on richness of tree genera, phylogenetic community diversity and structure. Then, we evaluated potential relationships between changes in phylogenetic diversity and the proliferation or declining of 59 tree genera that have significantly changed in importance values since forest isolation. Finally, we estimated the phylogenetic distribution of declining and increasing tree genera and discuss the implications of our results for the ecology and conservation of tropical tree communities in human-modified landscapes.

## Methods

### Study landscape

The Biological Dynamics of Forest Fragments Project is a 1,000-km^2^ experimental landscape in Central Amazonia. Within this landscape, nine forest fragments ranging from 1 to 100 ha in area were isolated from nearby intact forest during the early 1980s by clearing and burning the intervening vegetation to create cattle pastures. Some of the pastures have been abandoned and now support secondary forests. Detailed descriptions of the project, including its study design, fragment histories, the matrices of modified vegetation surrounding fragments, and the methods used for censusing and identifying trees are provided elsewhere [24].

Before fragment isolation, permanent 1-ha forest plots were established within each fragment and in eight comparable sites in nearby intact forest. The present study incorporates tree demography data from 40 1-ha plots distributed throughout the entire landscape, either along forest edges (plot center <100 m from the nearest edge) or in ‘intact’ forest interiors (170–3,000 m from the edge). After an initial, exhaustive inventory of tree communities, each plot was resampled after fragmentation at typical intervals of 4–6 years to assess tree mortality, growth, and the recruitment of new trees. Altogether, the fates of nearly 32,000 trees (dbh>10 cm) were followed for periods of up to 18 years (mean 14.7 years) [19]. All the tree specimens from the different censuses were identified together by collecting a voucher specimen for virtually every single tree. In the herbarium, all the voucher specimens were pooled into families (the initial family identification was made in the field) and then each specimen was identified to species or genus/morphospecies level.

In this study we used the same genus-level dataset presented in Laurance et al. [19], [32], which were mainly interested in describing broader taxonomic and ecological responses of tree communities to human disturbance in 40 1-ha BDFFP plots. We are aware that examining phylogenetic community structure at the genus level has shortcomings when compared with species-level analyses, as we cannot assume that shifts in tree genera are a proxy for shifts in tree species. However, in the absence of robust species-level phylogeny, the genus-level approach provides reliable information about lineages and can shed light on the structure of hyperdiverse communities such as ours. This approach has been used in other studies, including ant [33] and bacteria communities [34], and contributed considerably for the development of phylogenetic community ecology.

### Shifts in phylogenetic community structure and diversity

To evaluate shifts in tree communities we first listed all genera observed in the 40 1-ha plots. Altogether, 267 genera belonging to 62 families were recorded. This genera list was then assembled into a phylogeny (i.e. the regional phylogeny) using the phylomatic function of Phylocom 4.2 [35] and the maximally resolved supertree of angiosperms R20100701, available for free in the software. The regional phylogeny had branch lengths estimated with the bladj algorithm. For this, we used the node ages provided by Bell et al. [36] and further corrected for inconsistencies in syntax and nomenclature of internal nodes and the regional phylogeny following procedures described in Gastauer and Meira-Neto [37]. The resulting time-calibrated regional phylogeny was used in the subsequent analyses (see Appendix S1).

To examine shifts in phylogenetic community structure and diversity within the landscape, we calculated two intra-sample phylogenetic metrics for each community: mean phylogenetic distance (MPD) and net related index (NRI) (see Webb et al. [35] for a complete description of the metrics and Table S1 for raw data of tree plots). We used the argument ‘-a’ of comstruct function in Phylocom 4.2 to take into account the importance value of each taxon, estimated by the combination of basal area and relative abundance as described in Laurance et al. [19]. To be conservative, we avoided using metrics more sensitive to phylogenetic resolution at the tips of the phylogeny, such as MNTD and NTI, because the evolutionary relationships of many tropical genera within families are not resolved yet.

We treated MPD as the metric of phylogenetic diversity and NRI as the metric of phylogenetic structure. MPD was expressed in million years and represented the observed phylogenetic diversity of a given community. NRI, the measure of ‘standarzided effect size’ of phylogenetic community structure, was calculated comparing the observed MPD to 999 null communities generated by null model 0 of Phylocom 4.2. This model shuffles species labels across the entire phylogeny [35] and is more appropriate for temporal analyses than other null models (e.g. Phylocom model 2) because it maintains plot abundance distribution, plot species richness, occupancy rates, and conserves the spatial contagion of species (see Norden et al. [31], p.S74, for more details on the use of this null model for temporal analyses).

We then made before vs. after comparisons to assess whether average levels of tree-wide phylogenetic structure changed over time across forest sites. Positive trends of NRI indicate decrease in phylogenetic evenness or increase in phylogenetic clustering since forest isolation, whereas negative trends indicate the opposite. Because communities with similar values of NRI can differ in terms of absolute evolutionary diversity, we made similar comparisons using the observed values of MPD to estimate potential shifts in community phylogenetic diversity.

### Increasing and decreasing tree genera

Two previous studies [19], [32] identified 28 tree genera that are significantly increasing and 31 that are significantly decreasing in importance value throughout the landscape, totaling 59 genera that respond positively or negatively to human disturbance in demographic terms (see Table S2). The bulk of increasing genera is composed by fast-growing, small-seeded species typical of early to mid successional stages; 15 are thriving in edge-affected habitats [19] and 13 in the continuous forest [32]. The group of decreasing genera is mostly represented by slow-growing, large-seeded species typical of old-growth forests; 18 are declining in edge-affected habitats, six are reducing in the continuous forests and seven are declining in both habitats [19], [32].

To assess the relationship between the shifts in phylogenetic diversity and the proliferation or declining of these particular genera, we calculated for each tree community the relative change in MPD and mean importance value of the 59 tree genera that have significantly responded to human disturbance. The relative changes in MPD and mean importance value of genera were calculated as the difference between the final and the initial value, divided by the initial value. Negative relative changes in MPD indicate that tree community loses phylogenetic diversity over time, while positive changes indicate the opposite. If shifts in phylogenetic diversity were influenced by the proliferation of increasing genera, we would expect positive relationship between the gain of increasing genera and the loss of phylogenetic diversity. A similar positive relationship could arise if the negative shifts in MPD were affected by the loss of decreasing genera.

### Phylogenetic distribution of increasing and decreasing genera

To assess the phylogenetic distribution of declining and increasing tree genera, we estimated the degree of phylogenetic relationships for each group using NRI, as adopted elsewhere [31]. Both groups of genera, one containing 28 increasing genera and other 31 decreasing genera, were assumed to be a community drawn from the 267-genera regional pool, resulting in a value of NRI for group. According to the findings of Norden et al. [31], we expected that the group of increasing genera was more phylogenetically related than the group of decreasing ones (i.e. NRI_increasing_>NRI_decreasing_).

### Statistical analyses

We used linear mixed models to test for the effects of forest site, time since forest isolation and distance to the nearest forest edge on the richness of tree genera and phylogenetic community metrics. We set forest site (four levels: 1-ha [*n* = 4], 10-ha [*n* = 9], 100-ha forest fragments [*n* = 10] and continuous forest [*n* = 17]), time since forest isolation (first and last census), edge distance and the interaction between forest site×time as fixed effects. We included this interaction term to assess whether the magnitude of the shifts in phylogenetic metrics was greater in the smaller forest fragments (1-ha and 10-ha), where tree communities have changed suddenly since forest isolation [19]. Forest plot (*N* = 40), the subject in which tree censuses were done, was set as the random effect. We adopted the residual maximum likelihood method to separate the variance of fixed and random effects [38]. We used Pearson product-moment correlations to assess the relationship between the relative changes in MPD and mean importance value of decreasing and increasing genera. All analyses were performed in JMP 8 (SAS Institute Inc.).

## Results

**Figure 1:**
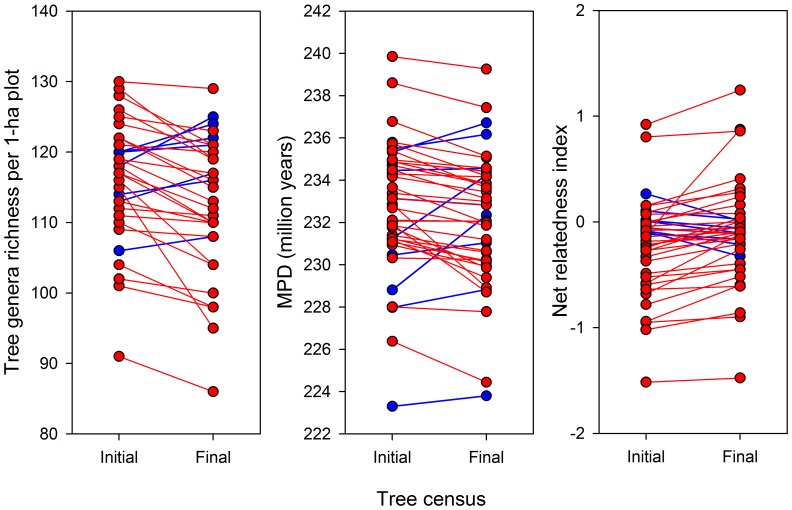
Tree genera richness and phylogenetic metrics of 40 1-ha tree communities in forest fragments and continuous forest in a 1000-km^2^ forest landscape of Central Amazonia, Brazil. Initial tree census refers to the first tree inventory carried out before forest isolation in early 1980's; final tree census refers to the last census available in our dataset (on average 14 years after the first census, see Methods). Red lines indicate loss of tree genera and phylogenetic diversity over time.

Genera richness decreased significantly from 116.2±1.2 genera/ha (mean ± SE) before fragmentation to 113.5±1.4 genera/ha after fragmentation (time effect in Table 1), but this reduction occurred regardless of whether plot was in forest fragments or continuous forest (interaction term not significant; Table 1). We observed a similar trend for MPD, which decreased slightly (<2%) but significantly over time and irrespective to plot location (Table 1; Fig. 1). Surprisingly, shifts in phylogenetic structure and diversity were not related with plot distance to the nearest edge (Table 1).

**Table 1:** Fixed effects of linear mixed models fitted for repeated measures of tree community attributes in a 1000-km^2^ forest landscape of Central Amazonia, Brazil.

Model terms	DF	*F*-ratio	*P*-value	Model Adj *R* ^2^(%)
Genera richness				
Site	3,35	2.04	0.126	91.9
Time	1,36	4.69	**0.037**	
Site×Time	3,36	1.76	0.172	
Edge distance	1,35	3.18	0.083	
MPD				
Site	3,35	2.46	0.079	94.2
Time	1,36	9.48	**0.004**	
Site×Time	3,36	1.39	0.261	
Edge distance	1,35	0.23	0.629	
NRI				
Site	3,35	3.59	0.023	93.3
Time	1,36	17.52	**<0.001**	
Site×Time	3,36	0.85	0.476	
Edge distance	1,35	0.14	0.711	

Site is represented by four levels: continuous forest, 1-ha, 10-ha, and 100-ha forest fragments. *N* = 40 tree communities, 1-ha each.

**Figure 2:**
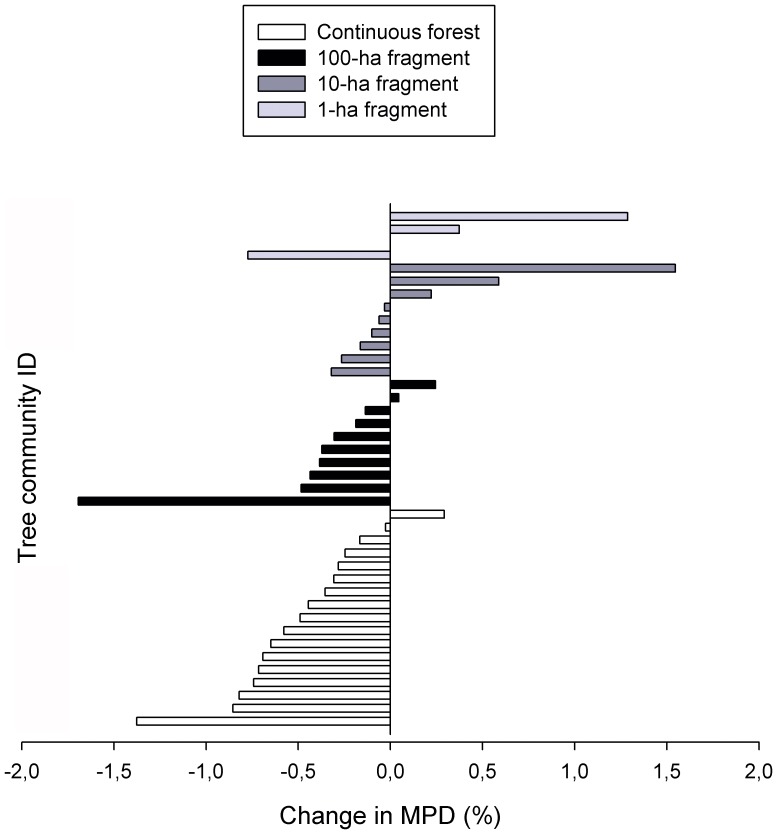
Relative change in mean phylogenetic distance across 40 tree communities in a 1000-km^2^ forest landscape of Central Amazonia, Brazil. Negative values indicate loss of phylogenetic diversity over time.

Thirty-two out of 40 communities (80%) experienced a decrease in MPD over the period of tree monitoring, as indicated by negative rates of change in MPD (Fig. 2). In these impoverishing tree communities, the loss of phylogenetic diversity was positively correlated with the increase in importance value of increasing genera (*r* = 0.38; *P*<0.05) and marginally correlated with the reduction in importance value of decreasing genera (*r* = 0.34; *P* = 0.059), suggesting that both the proliferation of increasing genera and the reduction of decreasing genera are partially underlying the loss of phylogenetic diversity at the study landscape.

Consistent with the reduction in genera richness and MPD, NRI increased after forest isolation, resulting in a 50%-decrease in tree-wide phylogenetic evenness of tree communities throughout the landscape (NRI_initial_ = −0.33 vs. NRI_final_ = −0.17). Furthermore, as expected, the group of 28 tree genera thriving in the landscape was more phylogenetically clustered than that of 31 declining genera (NRI_increasing_ = 1.56 vs. NRI_decreasing_ = 0.09).

## Discussion

In the last decades, the scientific community has invested immense efforts to anticipate the fate of tropical biodiversity as it becomes increasingly threatened by human-disturbances, such as habitat fragmentation, agricultural expansion and global warming [39], [40]. Much has been learnt from either theoretical models or empirical studies, at least at the species and community level, but the scarcity of long-term data and lack of reliable evolutionary information still limit our ability to understand the extent to which human-modified landscapes can operate as a repository for the tropical evolutionary heritage (see references [41]–[45] for contrasting views).

The present study bridges part of this knowledge gap by documenting shifts in the phylogenetic structure and diversity of tree communities inhabiting multiple sites in a human-modified landscape (see also Arroyo-Rodríguez et al. [46]). Despite the genus-level analyses and the great variation in the phylogenetic metrics across plots and sites, tree communities experienced significant reduction in tree genera richness, loss of phylogenetic diversity and reduction in phylogenetic evenness over time throughout the entire landscape. Such impoverishment was correlated with demographic shifts of particular genera, suggesting that the replacement of less related, slow-growing tree taxa by more related, fast-growing ones is partially underlying the erosion of evolutionary heritage at the landscape scale. Considering the long lifespan usually exhibited by tropical tree species, potentially exceeding several centuries [47], it is alarming that such shifts have already emerged within an experimental landscape exposed to habitat loss and fragmentation in the early 1980s.

Identifying the causes for the demographic and phylogenetic shifts we documented in this human-modified landscape is complex, especially across the supposed ‘intact’ continuous forest plots located from 170–3000 m from the nearest forest edge. A possibility is that these forest plots could be in a state of disequilibrium recovering from a large-scale past disturbance, leading to time-dependent shifts in community membership and phylogenetic community structure [14], [15], [31]. Major forest fires has been recognized as the only natural disturbance likely to operate at this scale [32], but charcoal and phytolith data suggest that the study area has been continuously forested for at least 4,500 years [48]. Another possibility is that old-growth forests would be responding to multi-decadal changes in rainfall, which affects forest productivity, species composition and possibly phylogenetic diversity. However, no trends in Central Amazonia rainfall were evident for the 1984–1999 period [32] or across preceding decades [49]. What has been documented so far is that our continuous forest plots are experiencing elevated mortality and turnover rates [50], leading to a significant decline in plot species richness [51] in parallel with the proliferation of fast-growing species [32], which also occur across all forest habitats [19], [26]. It has been hypothesized that the elevated mortality and recruitment are a consequence of rising atmospheric CO_2_ concentrations, which might stimulate plant growth and competition [32].

Independent findings in the Amazon and Brazilian Atlantic forests have provided evidence for a nonrandom taxonomic and functional impoverishment of tree communities inhabiting edge-affect habitats from local to regional scales [19]–[21], [25], largely due to shifts in physical conditions imposed by the creation of forest edges [25], [52]. Such a floristic/functional drift towards communities dominated by few disturbance-adapted fast-growing species ultimately results in increasing levels of biotic homogenization [23], [30] and loss of phylogenetic diversity along forest edges [16]. Although the phylogenetic shifts we documented in the study landscape were not related with plot distance to the nearest forest edge, it is not surprising that (1) tree communities experience phylogenetic diversity loss, clustering and possibly homogenization across multiple habitats and spatial scales, and (2) to some extent the evolutionary heritage of sensitive tropical forest biota will not be retained across human-modified landscapes [24].

Nonetheless, we cannot definitively discard the hypothesis that our findings are simply the outcome of stochastic variation in forest dynamics within the study landscape. This possibility is supported by the 17 reference plots in the continuous forest, which also showed significant temporal changes in genera richness and phylogenetic structure and diversity. We would need even longer tree monitoring, possibly over a century, to properly identify the causes of the taxonomic and ecological shifts that ultimately lead to the phylogenetic shifts at that spatial scale. Also, the long-term magnitude of tree community responses to habitat loss and fragmentation remains uncertain, precluding efforts to scale-up conclusions for other tropical forests. It has been proposed that taxonomic, functional and phylogenetic impoverishment of tree communities across multiple spatial scales are more likely to occur among those biota that have evolved and diversified in the presence of less intensive natural or human-related disturbance regimes, such as in Central Amazonia and the Brazilian Atlantic forests [53]–[55]. In contrast, forests that evolved under intense volcanic activity, frequent hurricanes, and extreme climates, such as some Mexican and Costa Rican forests, support a higher proportion of disturbance-tolerant species than do South America forests, and thus may be less susceptible to human disturbances [56]–[58]. Although the generality of the phylogenetic impoverishment we detected is unclear, it could negatively affect ecosystem productivity and stability and have broader impacts on coevolved organisms [8], [9]. Further studies should examine the generality of both patterns and processes proposed here.

## Supporting Information

Table S1 **Raw data of 40 1-ha tree plots, before and after fragmentation.**
(XLSX)

Table S2 **Increasing and decreasing tree genera at the study landscape in Central Amazonia, Brazil.**
(XLSX)

Appendix S1 **Regional phylogeny of the study landscape in Central Amazonia, Brazil.**
(TXT)
